# Genetic Diversity and Population Structure of *Saccharomyces cerevisiae* Strains Isolated from Different Grape Varieties and Winemaking Regions

**DOI:** 10.1371/journal.pone.0032507

**Published:** 2012-02-29

**Authors:** Dorit Schuller, Filipa Cardoso, Susana Sousa, Paula Gomes, Ana C. Gomes, Manuel A. S. Santos, Margarida Casal

**Affiliations:** 1 Centre of Molecular and Environmental Biology (CBMA), Department of Biology, University of Minho, Braga, Portugal; 2 BIOCANT, Centro de Inovação em Biotecnologia, Cantanhede, Portugal; 3 RNA Biology Laboratory, Department of Biology, University of Aveiro, Aveiro, Portugal; University of Nottingham, United Kingdom

## Abstract

We herein evaluate intraspecific genetic diversity of fermentative vineyard-associated *S. cerevisiae* strains and evaluate relationships between grape varieties and geographical location on populational structures. From the musts obtained from 288 grape samples, collected from two wine regions (16 vineyards, nine grape varieties), 94 spontaneous fermentations were concluded and 2820 yeast isolates were obtained that belonged mainly (92%) to the species *S. cerevisiae*. Isolates were classified in 321 strains by the use of ten microsatellite markers. A high strain diversity (8–43 strains per fermentation) was associated with high percentage (60–100%) of fermenting samples per vineyard, whereas a lower percentage of spontaneous fermentations (0–40%) corresponded to a rather low strain diversity (1–10 strains per fermentation).

For the majority of the populations, observed heterozygosity (*Ho*) was about two to five times lower than the expected heterozygosity (*He*). The inferred ancestry showed a very high degree of admixture and divergence was observed between both grape variety and geographical region. Analysis of molecular variance showed that 81–93% of the total genetic variation existed within populations, while significant differentiation within the groups could be detected. Results from AMOVA analysis and clustering of allelic frequencies agree in the distinction of genetically more dispersed populations from the larger wine region compared to the less extended region. Our data show that grape variety is a driver of populational structures, because vineyards with distinct varieties harbor genetically more differentiated *S. cerevisiae* populations. Conversely, *S. cerevisiae* strains from vineyards in close proximity (5–10 km) that contain the same grape variety tend to be less divergent. Populational similarities did not correlate with the distance between vineyards of the two wine regions. Globally, our results show that populations of *S. cerevisiae* in vineyards may occur locally due to multi-factorial influences, one of them being the grape variety.

## Introduction

Recent phylogenetic analyses of *Saccharomyces cerevisiae* strains have found that the species as a whole consists of both “domesticated” and “wild” populations. Although the genomes of most *S. cerevisiae* strains with disparate ecological and geographic sources are mosaics, genealogical relationships from DNA sequence diversity showed that domesticated strains derived from two independent clades, corresponding to strains from winemaking and sake (Japanese rice wine). “Wild” populations are mostly associated with oak trees, nectars or insects (e. g. *Drosophila* spp., honey bees and wasps) [1], [2], [3], [4], [5].

As reviewed by Martiny *et al*., [6], a growing body of evidence supports the idea that free-living microbial taxa exhibit biogeographic patterns. Bacterial species vary in abundance, distribution and diversity over various taxonomic and spatial scales, whereas genetic distance is correlated with geographic distance or environmental characteristics such as salinity, depth and latitude. To study the ecology and population dynamics of *S. cerevisiae* strains in both vineyards and wineries, numerous molecular methods were developed recently. Microsatellite analysis can be considered the method of choice for *S. cerevisiae* strain delimitation, allowing high-throughput and precise data generation. Besides, due to the high level of discrimination and unequivocal results, expressed as base pair number (or as repeat number), the generated data are suitable for computational population genetic analysis [7], [8], [9], [10]. Twelve highly polymorphic microsatellite loci were used to assess the genetic diversity among 651 *S. cerevisiae* strains from 56 worldwide geographical origins. The genotypes clustered in subgroups, according to the strain's technological use (i.e. bread, beer, wine, sake). Macrogeographical differentiation of strains from Asia, Europe and Africa accounted for 28% of the observed genetic variation, which suggests clonal reproduction and local domestication of natural strains originating from the same geographic area. [5]. Similar phylogenetic relationships related to technological applications were observed when clustering of *S. cerevisiae* strain was based on 32 single-nucleotide polymorphism markers [11] or amplified fragment length polymorphism (AFLP) analysis [12]. Recent studies with winemaking strains showed that populations are strongly structured [13] and that clonal reproduction is likely the main mating system with rare meiotic cycles, which is in agreement with a high percentage of inbreeding (80%). The transition between ‘domesticated’ and ‘natural’ isolates seems to be floating, and gene flow between subpopulations can be considered as significant [5], [13], [14], [15]. However, the forces shaping *S. cerevisiae* population structure are still poorly understood.

In our previous studies, we showed that microsatellites are informative markers for distinguishing populations from vineyards in very close geographical locations (50–100 km). Genetic differences and populational structures among *S. cerevisiae* strains derived from cumulative small microsatellite allele-frequency differences. Within a vineyard the strain's genetic divergence correlated with the distance between sampling points, suggesting a pattern of isolation-by-distance. However, geographical distance was not correlated with genetic proximity, pointing towards the involvement of other factors, for the differentiation of *S. cerevisiae* populations [8].

In this study we test the hypothesis that both geographical region and grape varieties are drivers of population structures of fermentative vineyard-associated *S. cerevisiae* strains. Grape samples of the nine most representative grape varieties were collected at the harvest time of two consecutive years in 16 vineyards from the Bairrada and Vinho Verde appellations of origin in Portugal (BAO and VAO, respectively). For populational analysis 288 samples were obtained, that concluded 94 spontaneous fermentations and 2820 yeast isolates were obtained that belonged mainly (94%) to the species *S. cerevisiae*, being classified in 321 strains using 10 polymorphic microsatellite markers.

## Results

### Wine regions, spontaneous fermentations and *S. cerevisiae* strain diversity

**Figure 1:**
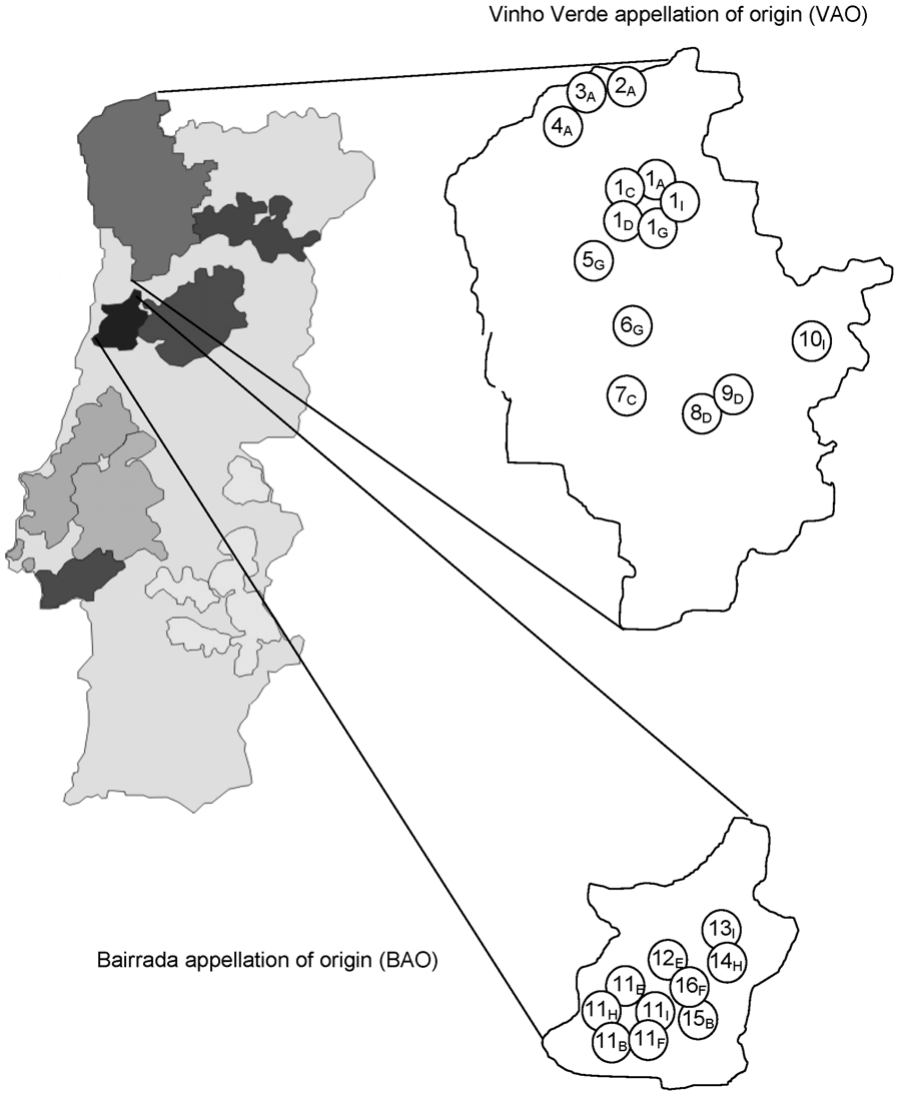
Geographic location of the vineyards 1–16 in the Vinho Verde and Bairrada apellations of origin (VAO and BAO), (1: Ponte da Barca; 2: Alvaredo; 3: Barbeita; 4: Longos Vales; 5: Ponte de Lima; 6: Amares; 7: Sousela; 8: São Tomé de Covelas; 9: Tresouras; 10: Ervedosa do Douro; 11: Quintã; 12: Cantanhede; 13: Mealhada; 14: Antes; 15: Outil; 16: Cerro). Subscript letters refer to the grape varieties that were cultivated in the vineyards and that were sampled within this study (A: Alvarinho; B: Aragonês; C: Arinto; D: Avesso; E: Baga; F: Bical; G: Loureiro; H: Maria Gomes; I: Touriga Nacional).

During the harvest time of two consecutive years, grape samples of representative grape varieties were collected in 16 vineyards (1–10 in the VAO and 11–16 in the BAO, Figure 1). The VAO is located in the north west of the country and constitutes the largest wine region in Portugal. The predominating white wine varieties are Alvarinho, Loureiro, Trajadura, Avesso, Arinto and Azal. The BAO region's most important red grape variety is Baga, producing full-coloured and acidic wines that are well-balanced and of great longevity. Maria Gomes is the predominant white grape variety, Touriga Nacional, Tinta Roriz, Arinto and Rabo de Ovelha are produced in smaller quantities.

In each wine region, five most representative grape varieties were collected (VAO: Alvarinho (A), Arinto (C), Avesso (D), Loureiro (G), Touriga Nacional (I); BAO: Aragonês (B), Baga (E), Bical (F) Maria Gomes (H), Touriga Nacional (I)), being Touriga Nacional shared by both wine regions. In vineyards 2–10 and 12–16, one single predominating grape variety was cultivated and collected. Vineyards 1 (VAO) and 11 (BAO) were chosen as reference vineyards, containing all five grape varieties of each region. With this approach, a total of 288 grape samples were collected for spontaneous fermentations. As detailed in Table 1, from 156 samples that were collected in the VAO region, 45 samples (28%) initiated a spontaneous fermentation and a total of 165 *S. cerevisae* strains were obtained (average: 3.6 strains per fermentation). In the vineyards of BAO, 132 grape samples were collected and 50 (38%) of spontaneous fermentations occurred, providing 156 *S. cerevisiae* strains (average: 3.1 strains per fermentation). The total yeast count (cfu in YPD medium, determined at the end of spontaneous fermentations) ranged between 1.0×10^6^ and 8.0×10^7^ cfu/ml of must. With a few exceptions, all isolates belonged to the species *S. cerevisiae* due to their inability to grow in a medium containing lysine as sole nitrogen source (data not shown), the amplification of *S. cerevisiae* specific PCR-based interdelta patterns and by the amplification of *S. cerevisiae* specific microsatellite loci (Table 2). No amplification was observed for the non-*Saccharomyces* species mentioned in Table 1 (not shown).

**Table 1:** Summary of the grape samples collected in the Bairrada and Vinho Verde regions, with indication of vineyards, grape varieties, sampling years, number of *S. cerevisiae* strains and Non-*Saccharomyces* species isolated.

Appellation of origin	Vineyard	Grape variety	Sampling year	Number of grape samples	Number of spontaneous fermentations	Number of *S. cerevisiae* strains	Non-*Saccharomyces* species (number of isolates)							
							*Candida glabrata*	*Candida zemplinina*	*Hanseniaspora uvarum*	*Hanseniaspora osmophila*	*Issatchenkia orientalis*	*Issatchenkia terricola*	*Kluyveromyces thermotolerans*	*Zygosaccharomyces bailii*
Vinho Verde	1	A	1	6	1	1	-	-	-	-	-	-	-	-
Vinho Verde	1	A	2	6	0	-	-	-	-	-	-	-	-	-
Vinho Verde	1	C	1	6	1	2	-	-	-	-	-	-	-	-
Vinho Verde	1	C	2	6	1	8	-	-	-	-	-	-	-	-
Vinho Verde	1	D	1	6	2	3	-	-	-	-	-	-	-	-
Vinho Verde	1	D	2	6	1	5	-	-	-	-	-	-	-	-
Vinho Verde	1	G	1	6	0	-	-	-	-	-	-	-	-	-
Vinho Verde	1	G	2	6	2	8	-	-	-	-	-	-	-	-
Vinho Verde	1	I	1	6	2	2	-	-	-	-	-	-	-	-
Vinho Verde	1	I	2	6	2	7	-	-	-	-	-	-	-	-
Vinho Verde	2	A	1	6	3	5	-	-	-	-	-	-	-	-
Vinho Verde	2	A	2	6	1	6	-	-	-	-	-	-	-	-
Vinho Verde	3	A	1	6	6	8	-	-	-	-	-	-	-	-
Vinho Verde	3	A	2	6	2	8	-	-	-	-	-	-	-	-
Vinho Verde	4	A	1	6	2	4	-	-	-	-	-	-	-	-
Vinho Verde	4	A	2	6	0	-	-	-	-	-	-	-	-	-
Vinho Verde	5	G	1	6	2	5	-	-	-	-	-	-	-	-
Vinho Verde	5	G	2	6	1	3	-	-	-	-	-	-	-	-
Vinho Verde	6	G	1	6	2	3	-	-	-	-	-	-	-	-
Vinho Verde	6	G	2	6	0	-	-	-	-	-	-	-	-	-
Vinho Verde	7	C	1	6	3	10	-	-	-	-	-	-	-	-
Vinho Verde	7	C	2	6	2	16	-	-	-	-	-	-	-	-
Vinho Verde	8	D	1	6	2	4	-	-	-	-	-	-	-	-
Vinho Verde	8	D	2	6	6	46	-	-	-	-	-	-	-	-
Vinho Verde	9	D	1	6	0	-	-	-	-	-	-	-	-	-
Vinho Verde	9	D	2	6	1	10	-	-	-	-	-	-	-	-
Vinho Verde	10	I	1	6	2	5	-	-	-	-	-	-	-	-
Vinho Verde	10	I	2	6	0	-	-	-	-	-	-	-	-	-
Bairrada	11	B	1	6	0	-	-	-	-	-	-	-	-	-
Bairrada	11	B	2	6	2	3	-	5	-	-	11	-	3	-
Bairrada	11	E	1	6	3	14	-	-	-	-	-	-	-	-
Bairrada	11	E	2	6	4	8	-	22	-	8	-	-	-	-
Bairrada	11	F	1	6	1	1	-	-	-	-	-	-	-	-
Bairrada	11	F	2	6	0	-	-	-	-	-	-	-	-	-
Bairrada	11	H	1	6	3	0	90	-	-	-	-	-	-	-
Bairrada	11	H	2	6	0	-	-	-	-	-	-	-	-	-
Bairrada	11	I	1	6	2	1	-	-	30	-	-	-	-	-
Bairrada	11	I	2	6	3	9	2	-	2	-	-	54	-	2
Bairrada	12	E	1	6	4	17	-	-	-	-	-	-	-	-
Bairrada	12	E	2	6	5	20	-	7	-	-	-	-	-	2
Bairrada	13	I	1	6	6	20	-	-	-	-	-	-	-	-
Bairrada	13	I	2	6	6	14	-	-	-	-	-	-	-	-
Bairrada	14	H	1	6	2	2	-	-	-	-	-	-	-	-
Bairrada	14	H	2	6	1	23	-	-	-	-	-	-	-	-
Bairrada	15	B	1	6	2	14	-	-	-	-	-	-	-	-
Bairrada	15	B	2	6	3	3	-	-	-	-	-	-	-	-
Bairrada	16	F	1	6	1	1	-	-	-	-	-	-	-	-
Bairrada	16	F	2	6	0	-	-	-	-	-	-	-	-	-

**Table 2:** Characteristics of all microsatellite loci that were used as genetic markers.

Microsatellite designation	Repeat	ORF or coordinates	Chromosome	Primers	Fluorochrome	Size (strain S288C)	N° of repeats (strain S288C)
ScAAT1	ATT	86 901–87 129	XIII	F: AAAAGCGTAAGCAATGGTGTAGAT	6-FAM	229	35
ScAAT1	ATT	86 901–87 129	XIII	R: AGCATGACCTTTACAATTTGATAT	6-FAM	229	35
ScAAT2	ATT	YBL084c	II	F: CAGTCTTATTGCCTTGAACGA	HEX	393	20
ScAAT2	ATT	YBL084c	II	R: GTCTCCATCCTCCAAACAGCC	HEX	393	20
ScAAT3	ATT	YDR160w	IV	F: TGGGAGGAGGGAAATGGACAG	6-FAM	268	23
ScAAT3	ATT	YDR160w	IV	R: TTCAGTTACCCGCACAATCTA	6-FAM	268	23
ScAAT4	ATT	431 334–431 637	VII	F: TGCGGAAGACTAAGACAATCA	TET	304	12
ScAAT4	ATT	431 334–431 637	VII	R: AACCCCCATTTCTCAGTCGGA	TET	304	12
ScAAT5	TAA	897 028–897 259	XVI	F: GCCAAAAAAAATAATAAAAAA	TET	231	13
ScAAT5	TAA	897 028–897 259	XVI	R: GGACCTGAACGAAAAGAGTAG	TET	231	13
ScAAT6	TAA	105 661–105 926	IX	F: TTACCCCTCTGAATGAAAACG	HEX	266	19
ScAAT6	TAA	105 661–105 926	IX	R: AGGTAGTTTAGGAAGTGAGGC	HEX	266	19
C4	TAA+TAG	110 701–110 935	XV	F: AGGAGAAAAATGCTGTTTATTCTGACC	TET	235	13+5
C4	TAA+TAG	110 701–110 935	XV	R: TTTTCCTCCGGGACGTGAAATA	TET	235	13+5
C5	GT	210250–210414	VI	F: TGACACAATAGCAATGGCCTTCA	TET	165	30
C5	GT	210250–210414	VI	R: GCAAGCGACTAGAACAACAATCACA	TET	165	30
YPL009c	TAA	*NFI*1	XV	F: AACCCATTGACCTCGTTACTATCGT	HEX	296	23
YPL009c	TAA	*NFI*1	XV	R: TTCGATGGCTCTGATAACTCCATTC	HEX	296	23
ScYOR267c	TGT	*HRK*1	XV	F: TACTAACGTCAACACTGCTGCCAA	6-FAM	186	21
ScYOR267c	TGT	*HRK*1	XV	R: GGATCTACTTGCAGTATACGGG	6-FAM	186	21

**Figure 2:**
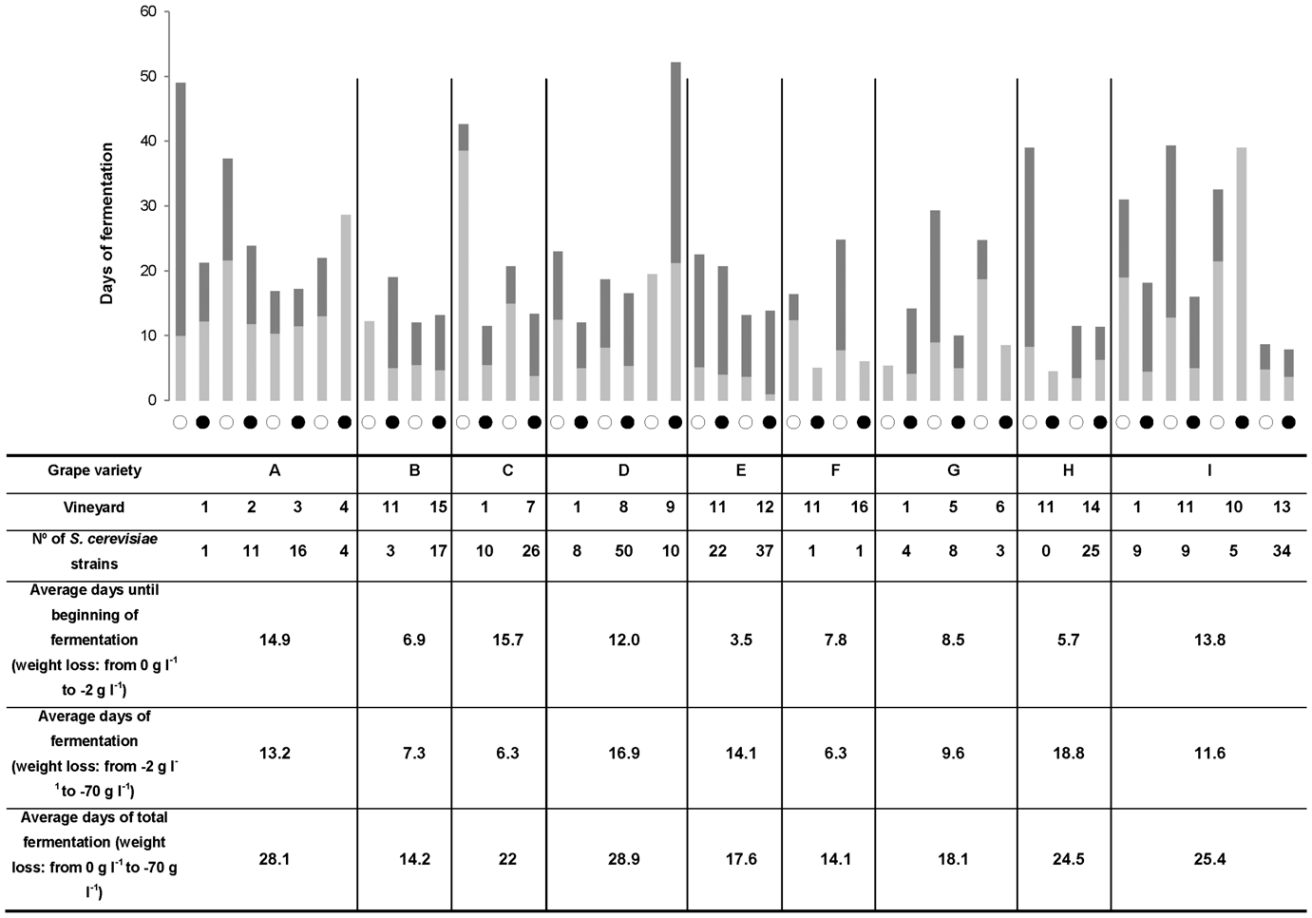
Summary of spontaneous fermentations. Bars indicate the average fermentation duration of must samples that underwent a spontaneous fermentation in each of the vineyards (1–16) and for all grape varieties (A–H) in sampling year 1 (open circles) and 2 (closed circles). The light grey part of the bars indicates the average number of days until the beginning of fermentation (lag time, corresponding to a weight loss of 2 gl^−1^), whereas the dark grey part indicates the average days of fermentation (corresponding to a weight loss from 2 gl-1 to 70 gl^−1^). The average number of *S. cerevisiae* strains from sampling years 1 and 2 is also indicated, as well as average lag and fermentation times for samples from all grape varieties. The number of spontaneous fermentations for each variety/vineyard combination is given in Table 1.

**Figure 3:**
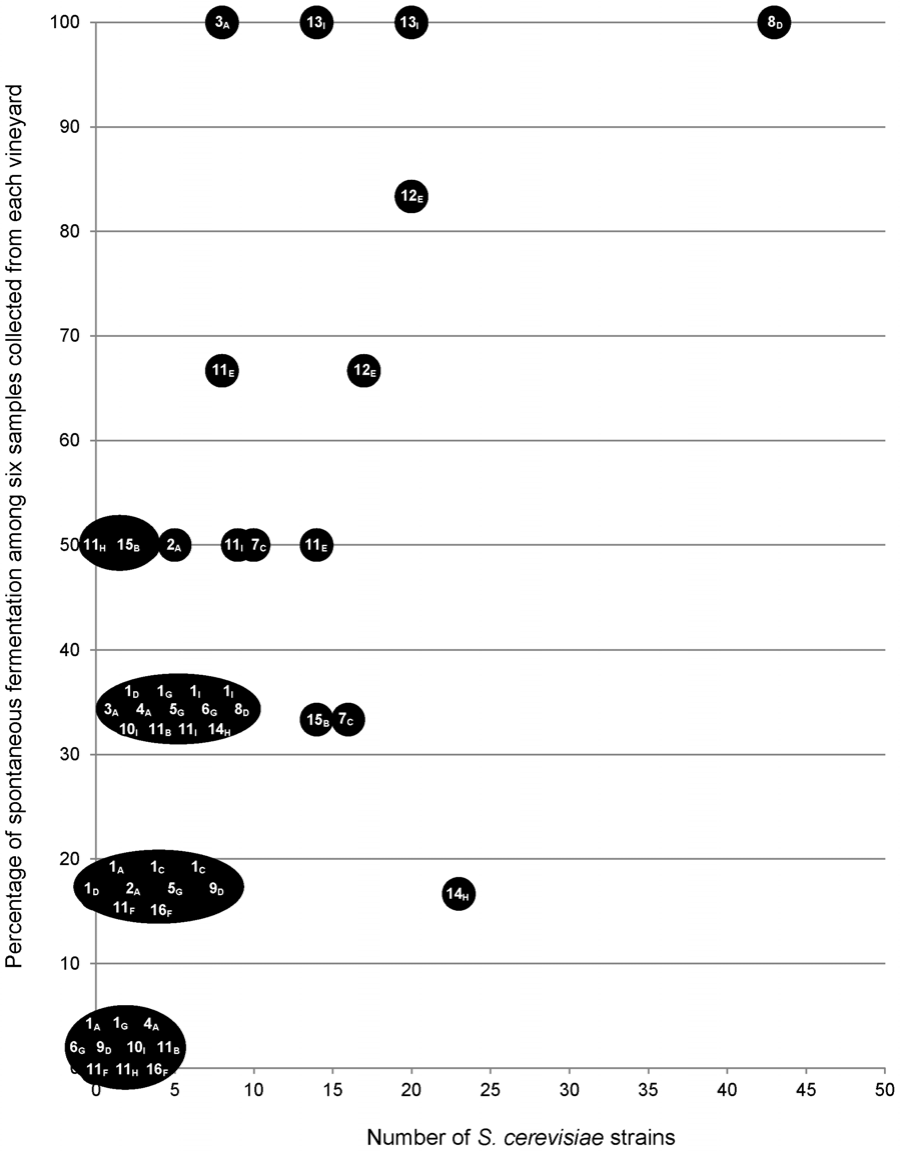
Diversity of *S. cerevisiae* strains from spontaneous fermentations carried out with musts from all vineyards (1–16) and all grape varieties (A–H; subscript letters) in sampling years 1 and 2, according to the percentage of spontaneous fermentations among six samples that were collected from each vineyard.

Figure 2 shows the main results regarding spontaneous fermentations and the isolated *S. cerevisiae* strains. The number of strains obtained from one vineyard in one sampling year was between 0–43 and 0–23 in the Vinho Verde and Bairrada region, respectively. Non-*Saccharomyces* species that are well-known for their occurrence in vineyards, were found in vineyard 11 in the samples from final stages of fermentations that were carried out using musts from the grape varieties Aragonês (B; year 2), Maria Gomes (H; year 1) and Touriga Nacional (I, year 1 and 2). The average duration until the beginning of fermentations (lag time, corresponding to a weight loss of 2 gl^−1^) was between 3.5 and 15.7 days (grape varieties H and C, respectively). The average fermentation period (corresponding to the weight loss from 2 gl^−1^ to 70 gl^−1^) was between 6.3 and 18.8 days (grape varieties F and H, respectively). Fermentations with grapes from the Bairrada region started within 6 days, whereas fermentation onset of musts collected in the Vinho Verde Region was much slower (14 days). However, the average fermentation duration was identical for the musts from both regions (12 days). Grape varieties that started fermentations most rapidly (E, H and B; 3.5, 5.7 and 6.9 days, respectively) correlated, by trend, with a higher number of *S. cerevisiae* strains (22+37, 25+3, 3+17 strains in year 1+2 of samples collected from grape varieties E, H and B, respectively). When the percentage of spontaneous fermentations among the six samples collected from each vineyard was compared with the number of isolated *S. cerevisiae* strains (Figure 3), it became evident that a high percentage (60–100%) of fermenting samples per vineyard was associated with higher strain diversity (between 8 and 43 strains per vineyard), whereas low percentage of spontaneous fermentations (0–40%) was associated with rather low strain diversity (between 1 and 10 strains per vineyard). In vineyards 12, 13 and 11 (grape variety E), the high percentage of spontaneous fermentations and strain diversity was observed in both years.

### Populational analysis of *S. cerevisiae* strains from different grape varieties in the Bairrada and Vinho Verde appellations of origin

**Figure 4:**
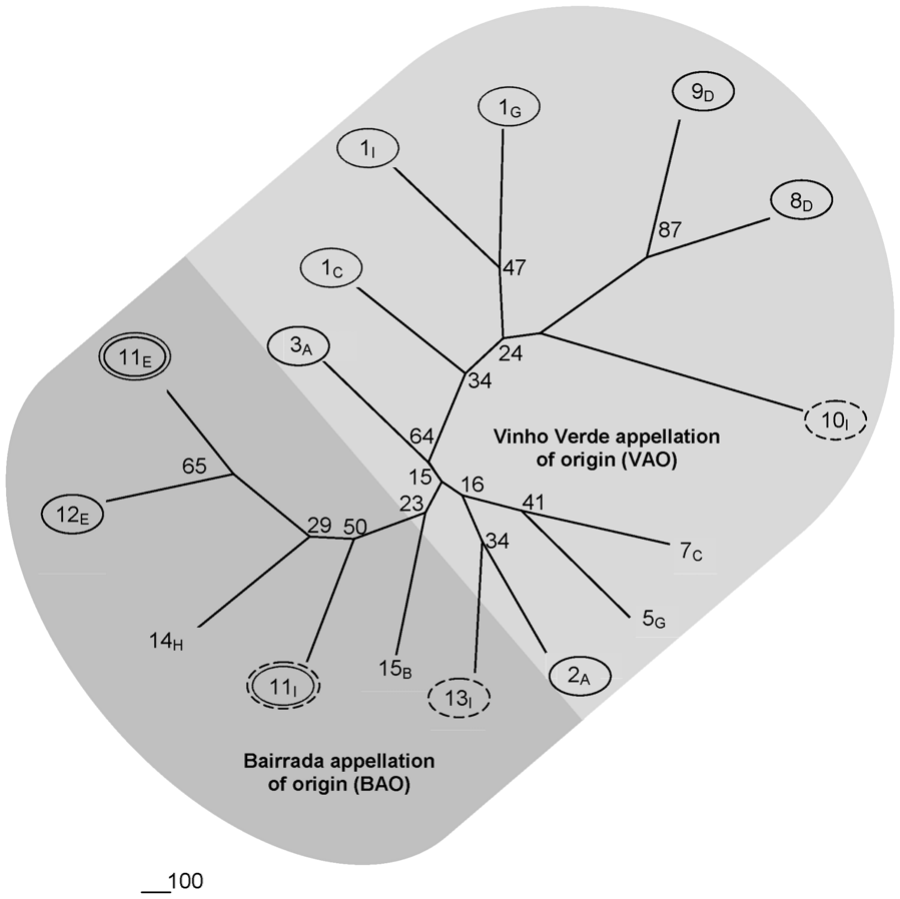
Consensus tree of 16 *Saccharomyces cerevisiae* populations (285 strains) from the Vinho Verde and Bairrada wine regions, shown as a neighbor-joining tree of allelic frequencies. Numbers on nodes are percentages of bootstrap values out of 1000. Populations from the same grape variety (8_D_/9_D_; 11_E_/12_E_; 2_A_/3_A_) are indicated by full circles, whereas groups of strains from the same vineyard (1_I_/1_C_/1_G_; 11_I_/11_E_) and from grape variety I (10_I_/11_I_/13_I_, collected in vineyards from both winemaking regions), are indicated by dotted and dashed circles, respectively.

The isolated *S. cerevisiae* strains were unique for each vineyard and were also not re-isolated in consecutive years. In addition, none of the strains corresponded to the commercial strains that were used by the wineries in the last few years. The extent of genetic divergence among *S. cerevisiae* populations from different grape varieties and sampling sites was examined by clustering allelic frequencies. Figure 4 shows the tree obtained by the neighbour-joining method. This analysis included strains from both sampling years and only vineyards were included from where at least five *S. cerevisiae* strains were obtained to provide a more representative quantification of allelic frequencies.

The highest bootstrap support was found for *S. cerevisiae* populations from grape variety E in the vineyards 11 and 12 (BAO), as well as grape variety D in vineyards 8 and 9 (VAO), which were 5–10 Km apart. These populations were also well separated from other groups. Conversely, the *S. cerevisiae* strains from different grape varieties in the same vineyards (11_E_ and 11_I_; 1_C_, 1_I_ and 1_G_) were much more divergent. The grape variety Touriga Nacional (I) is cultivated in most of Portuguese winemaking regions and was therefore included as a reference in our approach. *S. cerevisiae* populations from these grapes obtained in vineyards 1, 10, 11 and 13 were unresembling. In summary, we can assert that populational structures prevail according to winemaking regions, whereas *S. cerevisiae* populations are most similar in vineyards in close vicinity (at least up to 10 km) where the same grape varieties were cultivated.

**Figure 5:**
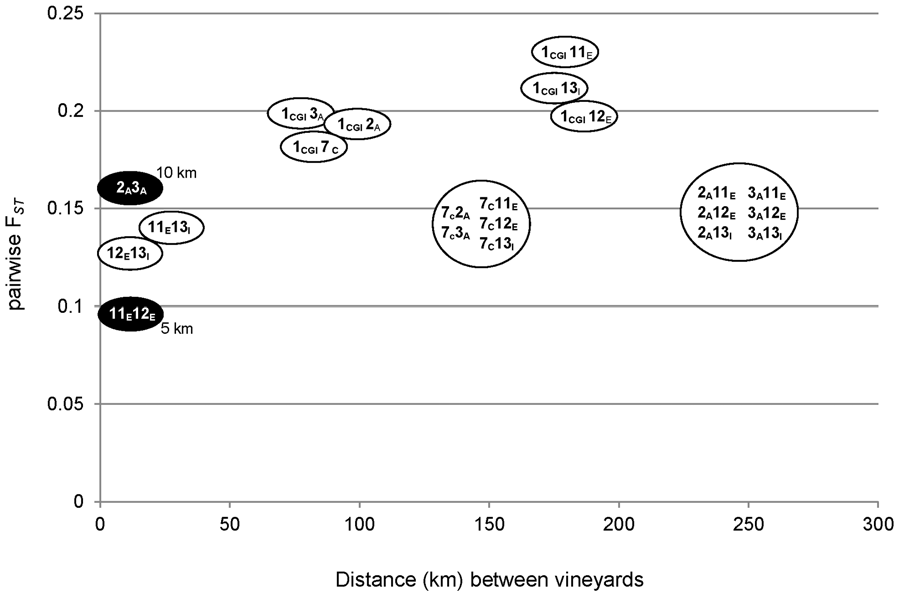
Correspondence analysis between geographic distance and population differentiation (F*_ST_*) for pair wise comparisons of *S. cerevisiae* populations from vineyards 1, 2, 3, 7, 11, 12, 13 and grape varieties A, C, D, E and I. Comparisons between vineyards with the same grape variety are shown in black ovals. All comparisons are statistically significant (P_(random value<observed value)_<0.000001).

For the majority of the populations, observed heterozygosity (*Ho*) was about two to five times lower than expected heterozygosity (*He*) for the ten loci analyzed (Table S1). Populations from vineyards 8 and 9 showed a higher heterozygosity than the expected values (*Ho*/*He*>1) for six microsatellites. The average of F*_IS_* values over all loci was rather high for most groups (mean of 0.61), pointing towards inbreeding as the predominating reproductive mechanism. The pattern and degree of populational divergence in the ten nuclear microsatellites among subpopulations was estimated by F*_ST_* determination over all loci by AMOVA analysis, as shown in Table 3 and Figure 5. For this analysis, only *S. cerevisiae* populations were included that consisted of at least 5 isolates (per sampling year, grape variety and vineyard). The contribution of variation within the populations defined was always high, ranging from 81 to 93%. For the analysis between wine regions, vineyards and grape varieties, the assemblage of several populations was considered as a group, indicated by parenthesis in Table 3. For all analyses, differences within groups constituted 7 to 16%, whereas differences among groups constituted only up to 7% of variation. F*_ST_* values ranged between 0.07 and 0.19, corresponding to a moderate (0.05–0.15) to great (0.15–0.25) genetic differentiation [16]. The highest F*_ST_* value was obtained for the comparison of vineyards 1 and 11 that contained multiple grape varieties and that are located at a distance of 180 km. This value decreased to 0.133 and 0.145 when all populations from VAO and BAO were compared, including or not the populations from vineyards 1 and 11, respectively. In agreement with the data presented in the previous section, *S. cerevisiae* populations were less divergent in the smaller BAO, where F*_ST_* values ranged between 0.067 and 0.120, whereas the corresponding values for the larger VAO region were between 0.157 and 0.173.

**Table 3:** AMOVA analysis, F*_ST_* values and distribution of variance components (%) among groups (AG), among populations within groups (APWG), and within populations (WP) based on microsatellite data of *S. cerevisiae* populations obtained from the indicated vineyards and grape varieties.

Wine region	Source of variation	Combination of groups of vineyards and grape varieties	Percentage of variation (AG)	Percentage of variation (AGWP)	Percentage of variation (WP)	F*_ST_*	P (r<o)
VAO and BAO	All vineyards	(1_C_ 1_G_ 1_I_ 2_A_ 3_A_ 5_G_ 7_C_ 8_D_ 9_D_ 10_I_) ⇔ (11_E_ 11_I_ 12_E_ 13_I_ 14_H_ 15_B_)	3.52	10.94	85.54	0.145	<0.000001
VAO and BAO	Vineyards with single grape varieties	(2_A_ 3_A_ 5_G_ 7_C_ 8_D_ 9_D_ 10_I_) ⇔ (12_E_ 13_I_ 14_H_ 15_B_)	3.20	10.08	86.72	0.133	<0.000001
VAO and BAO	Vineyards 1 and 11	(1_C_ 1_G_ 1_I_) ⇔ (11_E_ 11_I_)	6.32	12.62	81.06	0.189	<0.000001
VAO and BAO	Grape variety I	(1_I_ 10_I_) ⇔ (11_I_ 13_I_)	−0.80	16.09	84.71	0.153	<0.000001
VAO	Grape varieties	(2_A_ 3_A_) ⇔ (1_C_ 7_C_) ⇔ (8_D_ 9_D_) ⇔ (1_G_ 5_G_) ⇔ (1_I_ 10_I_)	2.77	12.94	84.29	0.157	<0.000001
VAO	Vineyard 1 and other vineyards/grape varieties	(1_C_ 1_G_ 1_I_) ⇔ (2_A_ 3_A_ 5_G_ 7_C_ 8_D_ 9_D_ 10_I_)	3.59	13.71	82.69	0.173	<0.000001
VAO	Vineyard 1 and other vineyards/grape varieties	(1_C_ 1_G_ 1_I_) ⇔ (12_E_ 13_I_ 14_H_ 15_B_)	6.07	9.69	84.25	0.157	<0.000001
VAO	Vineyard 1 and other vineyards/grape varieties	(1_C_ 1_G_ 1_I_) ⇔ (11_E_ 11_I_ 12_E_ 13_I_ 14_H_ 15_B_)	7.14	8.28	84–58	0.154	<0.000001
BAO	Grape varieties	(11_E_ 12_E_) ⇔ (11_I_ 13_I_)	1.95	10.19	87.85	0.120	<0.000001
BAO	Vineyard 11 and other vineyards/grape varieties	(11_E_ 11_I_) ⇔ (2_A_ 3_A_ 5_G_ 7_C_ 8_D_ 9_D_ 10_I_)	3.73	11.18	85.09	0.150	<0.000001
BAO	Vineyard 11 and other vineyards/grape varieties	(11_E_ 11_I_) ⇔ (12_E_ 13_I_ 14_H_ 15_B_)	−0.54	7.24	93.30	0.067	<0.000001
BAO	Vineyard 11 and other vineyards/grape varieties	(11_E_ 11_I_) ⇔ (1_C_ 1_G_ 1_I_ 2_A_ 3_A_ 5_G_ 7_C_ 8_D_ 9_D_ 10_I_)	2.82	13.84	83.33	0.167	<0.000001

All comparisons are statistically significant (P_(random value<observed value)_<0.000001).

To further investigate associations between genetic differentiation and geographic distance, pair wise vineyard comparisons were performed according to their geographic distance (Figure 5). The genetic divergence was again highest when population 1_CGI_ was compared with other populations. In this case, the highest F*_ST_* values (0.20–0.23) were obtained when 1_CGI_ was compared with the more distant BAO populations (11_E_, 12_E_ and 13_I_), whereas lower values (F*_ST_* 0.18–0.20) were found when 1_CGI_ was checked against the closer VAO populations 2_A_, 3_A_ and 7_C_, suggesting a geographic correlation. However, this was not observed for the majority of the remaining comparisons, where F*_ST_* values ranged between 0.13–0.16, independent of the geographic distance. The lowest F*_ST_* value of 0.1 was found for populations from grape varieties E in the vineyards 11 and 12, located at a distance of 5 km. However, a strict correlation between grape variety and geographic proximity cannot be assumed because populations from vineyards 2 and 3, where variety A was cultivated were more divergent (F*_ST_* 0.16) than populations from distinct grape varieties in vineyards 11, 12 and 13, that were located from each other at similar distances. These data show that the grape variety can be in fact a driver of populational structures because vineyards with distinct varieties (1 and 11) harbor genetically more differentiated populations, whereas vineyards with the same grape varieties in close proximity (11 and 12) contain less divergent groups of strains.

**Figure 6:**
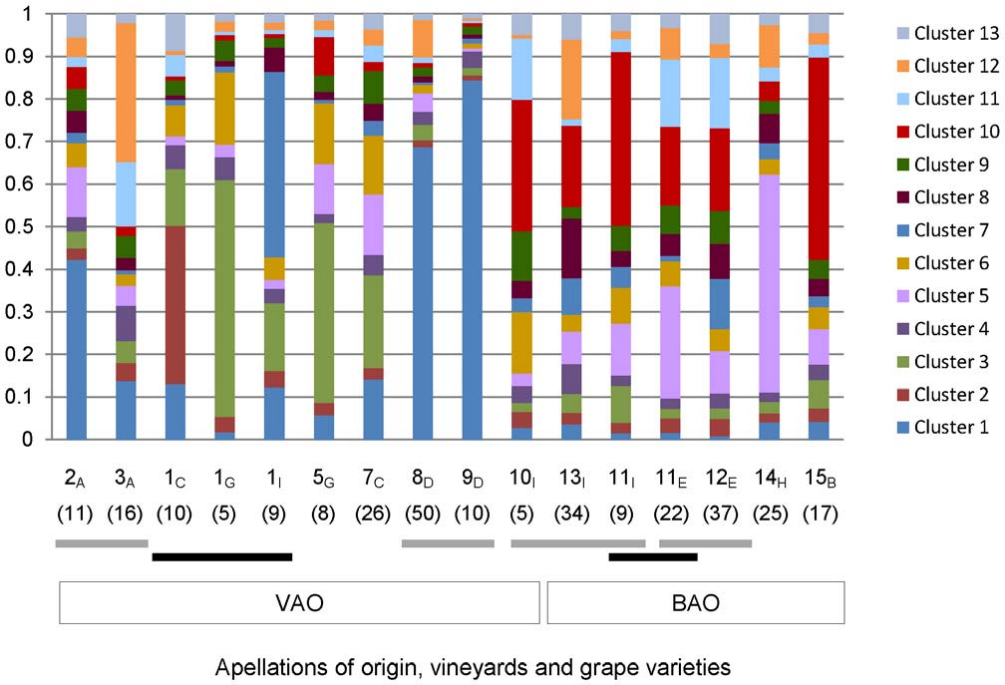
Results of InStruct analysis. Optimal alignments of replicate clusters were determined by the CLUMPP software. Each population is represented by a vertical bar partitioned into colored segments according to the probability of belonging to one of the 13 color-coded genetic clusters. Numbers in parenthesis correspond to the numbers of strains. Grey and black bars label *S. cerevisiae* populations from the same grape varieties and vineyards, respectively.

The Bayesian cluster estimation of population structure due to inbreeding was done using the software Instruct that determined the optimal number of 13 clusters. Each run used a burn-in period of 200,000, followed by 100,000 iterations. Ten replicate runs were performed and the CLUMPP software was used for finding optimal alignments of replicate cluster analyses of the same data, using the greedy algorithm that computed a symmetric similarity coefficient of 0.89. The inferred ancestry of populations is given in Figure 6. *S. cerevisiae* populations were distinguished by a considerable degree of admixture. Deeper divergence was observed between both geographic regions and grape varieties, being more evident in the VAO region which is in agreement with AMOVA analysis. Populations from vineyards 8 and 9, that shared a heterozygous excess (*Ho*/*He*>1) for six microsatellites loci, can be clearly distinguished. Clusters 1 and 3 were more represented in VAO populations, whereas clusters 5 and 10 were more predominant in BAO populations. Populations from multiple grape varieties in vineyards 1 and 11 (black bars in Figure 6) were more diverse in vineyard 1 compared to vineyard 11, in agreement with previously presented data.

## Discussion

Vineyards are an important yeast ecosystem. *S. cerevisiae* occurs in extremely low number on healthy undamaged grape berries (<0.1%) or soils [17], [18], [19], while damaged grapes provide inocula of 10^2^–10^3^cells/ml must [20]. A plethora of studies documented the occurrence and dynamics of *S. cerevisiae* in many wine regions in France [17], [21], [22], [23], [24] Spain [25], [26], [27], [28], [29], [30], Portugal [8], [31], Germany, Switzerland and Austria [32], [33], Italy [34], [35] Hungary, Bosnia and Herzegovina [36], [37], [38], Greece [39], South Africa [40], [41], [42], New Zealand [13], [14], Chile and Peru [15], Argentina [43], India [44] and China [45]. While most of these studies are rather descriptive in terms of yeast diversity, recent ecological studies show relationships between yeast communities and agricultural practices such as the farming (organic *versus* conventional) and floor management systems [46], [47].

In our previous studies we showed that within a vineyard the genetic divergence of *S. cerevisiae* strains correlated with the distance between sampling points, suggesting a pattern of isolation-by-distance. However, this relationship was not found for larger geographical distances, pointing towards the involvement of other factors such as the grape variety, which we evaluate within the present study. We highlight that *S. cerevisiae* isolates were obtained after enrichment through must fermentation and therefore may not accurately represent vineyard populations. Our experimental approach is therefore an acceptable compromise that allows for estimation of population composition, but does not provide a precise description in terms of relative strain abundance in nature.

Fermented musts from the grape varieties C, D and E (but also others such as varieties B, H and I in vineyards 15, 14 and 13, respectively) showed a notable strain diversity, which seems to be correlated with the percentage of spontaneous fermentations in a vineyard. Contrarily to the view that strains compete for nutrients under stressful fermentative conditions, it was surprising to find numerous strains at the end of the fermentations, suggesting a cooperative effect that may guarantee efficient fermentation when strain diversity is rather high. The faster fermentation onset observed for several grape varieties might also be related with a more favorable nutritional composition of the grapes. Spontaneous fermentations can be considered as complex multifactorial process, where strain diversity is one variable for a rapid onset, while the grape variety appears to be also relevant.

The occurrence and survival of *S. cerevisiae* in vineyards depend on climatic factors [19], [48] or viticulture practices [46], [47], [49], [50]. These were very similar in almost all vineyards studied (data not shown) with the exception of vineyard 8, where biodynamic organic farming is being practiced for several years according to the anthroposophy of Rudolf Steiner (1861–1925). This vineyard had a very high *S. cerevisiae* strain abundance, elevated percentage of spontaneous fermentations and low fermentative lag time compared to the closely located (10 km) vineyard 9, where the same soil, microclimatic conditions grape variety occurred. This result is in agreement with recent research showing that phytosanitary treatment has an impact on grape associated biodiversity [46]. Further studies on this topic are required, considering in particular that the production of organic wines relies solely on the yeast communities on the grape surfaces and winery environments.

Microsatellite typing of the chosen loci (Table 2) followed by allelic analysis permitted a fine populational screen, and revealed deeper insight into the biogeography of *S. cerevisiae* strains, even within geographically close regions. The isolated *S. cerevisiae* strains were unique for each vineyard and were also not re-isolated in consecutive years. However, in our previous research we found strains with a wider temporal and spatial distribution [8]. This difference could be explained by the fact that ten microsatellite loci were included in the present study, whereas our previous work relied on the analysis of six loci. Isolates with identical alleles for six loci might not share alleles of the remaining four loci and might therefore be considered as different strains with increasing number of analyzed loci. Genetic differences among *S. cerevisiae* populations derived mainly from gradations in allele frequencies rather than from distinctive “diagnostic” genotypes. These markers are useful for unambiguous populational analysis, but it needs to be considered that sub-strain level discrimination may occur due to their relative high mutation rate. Clonal expansion with some cycles of homothallic self-mating is considered to be the most likely reproduction in yeasts, generating the high observed homozygosity and very structured populations due to inbreeding or genome renewal [51]. The determined F*_IS_* values suggest that Portuguese yeast populations are inbred, which is in agreement with previous results obtained with strains from Chile and New Zealand, where F*_IS_* values ranged between 0.4–0.75 [13], [14], [15]. Heterozygote reduction can be explained by mitotic recombination, gene conversion during asexual reproduction or by the presence of null alleles that arise when mutations prevent primer annealing. Genetic differentiation may result from natural selection favoring different genotypes in different subpopulations, but also from random processes in the transmission of alleles from one generation to the next or from stochastic differences in allele frequency among the initial founders of the subpopulations. Populations from vineyard 8 and 9 showed a low genetic variation and seemed to evolve towards increased heterozygosity at multiple loci such as a clonal population evolving only under mutation. The allelic combinations in these groups of strains were very similar and varied frequently by just one microsatellite repeat among alleles for all loci. These populations might have lost genetic variability after a bottleneck. In such a case, variability starts to increase due to new mutations as soon as the population size becomes larger, whereas the average number of alleles per locus might increase faster than the average heterozygosity after the bottleneck. In alternative, strains of these populations could be affected by microsatellite instability associated with defective DNA mismatch repair as described for human malignancies.

Various approaches were used to determine the genetic structure of *S. cerevisae* populations from different vineyards and grape varieties. Results from F*_ST_* analysis and clustering of allelic frequencies agree in the distinction of genetically more dispersed populations from the larger VAO compared to the BAO region. Our data indicate that the grape variety can be a driver of populational structures because populations associated with different grape varieties from vineyards 1 and 11 were genetically more divergent than populations obtained from the same grape variety in vineyards in close locations (11_E_–12_E_ (5 km), 8_D_–9_D_ (10 km)). Comparison of strains from variety A in the close vineyards 2 and 3 (10 km) revealed a higher F*_ST_* value, but these populations were still less differentiated than the ones obtained from vineyard 1. A correlation between genetic and geographical distances was only evident when the more divergent populations from vineyard 1 were compared to other groups of strains. The higher genetic differentiation of yeasts from the experimental vineyard 1 may be attributed to the fact that it contains ten different grape varieties in larger quantities and 152 varieties of a clonal ampelographic collection, that were introduced three to four years before our study was initiated. The inferred ancestry of populations support strong admixture whereas divergence was again observed between both geographic regions and grape varieties. Interestingly population 10_I_ from VAO seemed more related with populations from BAO when the inferred ancestry was analyzed, This is one of the few varieties that is used in all Portuguese winemaking regions and might have been introduced in the VAO together with the yeast strains as a commensal member of grapevine flora, as previously suggested by Legras [5].

Recent studies showed that *S. cerevisiae* strains have been globally dispersed by humans, supporting the importance of geography in shaping *S. cerevisiae*'s population structure [5], [14]. However, for close geographical locations this association is not evident. Globally, our results show that a correlation between genetic distance and grape variety can arise. Local populations of *S. cerevisiae* in vineyards occur due to multi-factorial influences, being the grape variety one of them. These findings are in agreement with a recent report about distinctive non-*Saccharomyces* yeast populations occurring on different grape varieties in the same vineyard [46]. It is desirable to extend these studies to table-3-captionenvironmental genomics approaches regarding the abundance, distribution and diversity of yeasts in natural environments. Such data may also contribute to improved vineyard management and the elucidation of the role of yeast communities from specific grapevines to the outcome of spontaneous or industrial fermentations.

## Materials and Methods

### Sampling

The sampling plan included a total of 16 vineyards (ten in the Vinho Verde and six in the Bairrada region) as shown in Figure 1. The grape varieties cultivated in each vineyard correspond to the recommended varieties for both winemaking regions (Vinho Verde: Alvarinho, Avesso, Arinto, Loureiro; Bairrada: Aragonês, Baga, Bical and Maria Gomes). Besides, grapes of the Touriga Nacional variety were sampled, which is common to most of the Portuguese winemaking regions. In each vineyard, six sampling points were defined according to the size of the vineyard. Healthy and undamaged grape samples were collected a few days before the harvest, in two consecutive years. Grapes were not always collected from the same rootstock, but from the same area (±1–2 m). Vineyards 2–10 (VAO) and 12–16 (BAO) contained mainly one predominant grape variety. In addition, one vineyard was chosen in each region, where multiple grape varieties were cultivated (vineyard 1: Alvarinho, Avesso, Arinto, Loureiro, Touriga Nacional; vineyard 11: Aragonês, Baga, Bical, Maria Gomes and Touriga Nacional) to evaluate associations between the *S. cerevisiae* populations and the grape variety. All necessary permits were obtained for the described field studies, the owners of the vineyards agreed with the collection of grape samples and the sampling plan.

### Fermentation and strain isolation

From each sampling point, approximately 2 kg of undamaged and healthy grapes were aseptically collected and the extracted grape juice was fermented at 20°C in small volumes (500 ml). Fermentation progress was monitored by daily weight determinations. When must weight was reduced by 70 g/l, corresponding to the consumption of about 2/3 of the sugar content, diluted samples (10^−4^ and 10^−5^) were spread on YPD plates (yeast extract, 1% w/v, peptone, 1% w/v, glucose 2% w/v, agar 2%, w/v), and 30 randomly chosen colonies were collected after incubation (2 days, 28°C). The isolates obtained throughout this work were stored in glycerol (30%, v/v) at −80°C.

### Molecular analysis

Yeast cells were cultivated in 1 ml YPD medium (36 h, 28°C, 160 rpm). DNA isolation was performed as previously described [9].

In a preliminary approach, all isolates were analysed by interdelta sequence typing [9], [52]. One representative strain from each group of isolates with identical interdelta amplification patterns was further analysed by the microsatellite loci summarized in Table 2, using previously described PCR mixtures and amplification conditions [7], [8], [9], [10]. Isolates that showed no interdelta pattern and failed to amplify ten microsatellite loci were considered to belong to non-*Saccharomyces* species. These species were identified by restriction analysis of the ribosomal internal transcribed spacer (ITS) region as previously described [53]. The ITS region of representative isolates from each restriction pattern was sequenced for further confirmation. According to our (unpublished) results, the primer pairs used for amplification of microsatellite loci are predominantly specific for *S. cerevisiae* and fail to amplify all of the corresponding homologous loci in sibling species such as *S. bayanus* and *S. paradoxus* that can be found occasionally in winemaking environments. We therefore consider that these sibling species did not occur in our spontaneous fermentations.

### Data analysis

Based on the genome sequence for strain S288C (SGD database, http://genome-www.stanford.edu.saccharomyces) and the results obtained for the size of microsatellite amplicons of this strain, the number of repeats for all alleles was calculated. Allelic frequencies, observed and expected heterozygosity, F*_IS_* determinationas well as AMOVA analysis was performed by the software Arlequin 3.11 [54]. An allelic frequencies matrix, based on Euclidean distance was computed and clustered by the neighbour joining algorithm using the program PowerMarker [55]. The validity of nodes was obtained with the Consens program (Phylip3.69 package). Bayesian individual clustering of 16 populations was performed with the software INSTRUCT [56], which infers population structure and selfing rates at the population level. Assuming a number of clusters from K = 1–20, the most likely number of clusters was 13. Following, the program was run with 10 chains (200,000 iterations with 100,000 burn-in steps) and the optimal alignments of 10 replicate cluster analyses was determined by the CLUMPP software [57] using the greedy algorithm.

## Supporting Information

Table S1 **Observed (** ***Ho*** **) and expected (** ***He*** **) heterozygosity for** ***S. cerevisiae*** **populations from vineyards 1–15 and grape varieties A–I.** The ratios between observed (*Ho*) and expected (*He*) heterozygosity are indicated by underlined bold letters (*Ho*/*He*>1) and underlined letters (0.5>*Ho*/*He*>1). For the remaining fields the ratio was <0.5.
(TIF)
