# Supplementary figures and images for: Genetic Diversity and Population Structure of Saccharomyces cerevisiae Strains Isolated from Different Grape Varieties and Winemaking Regions
Source: PLoS One. 2012 Feb 29;7(2):e32507. doi: 10.1371/journal.pone.0032507 (PMC3290581; doi:10.1371/journal.pone.0032507)

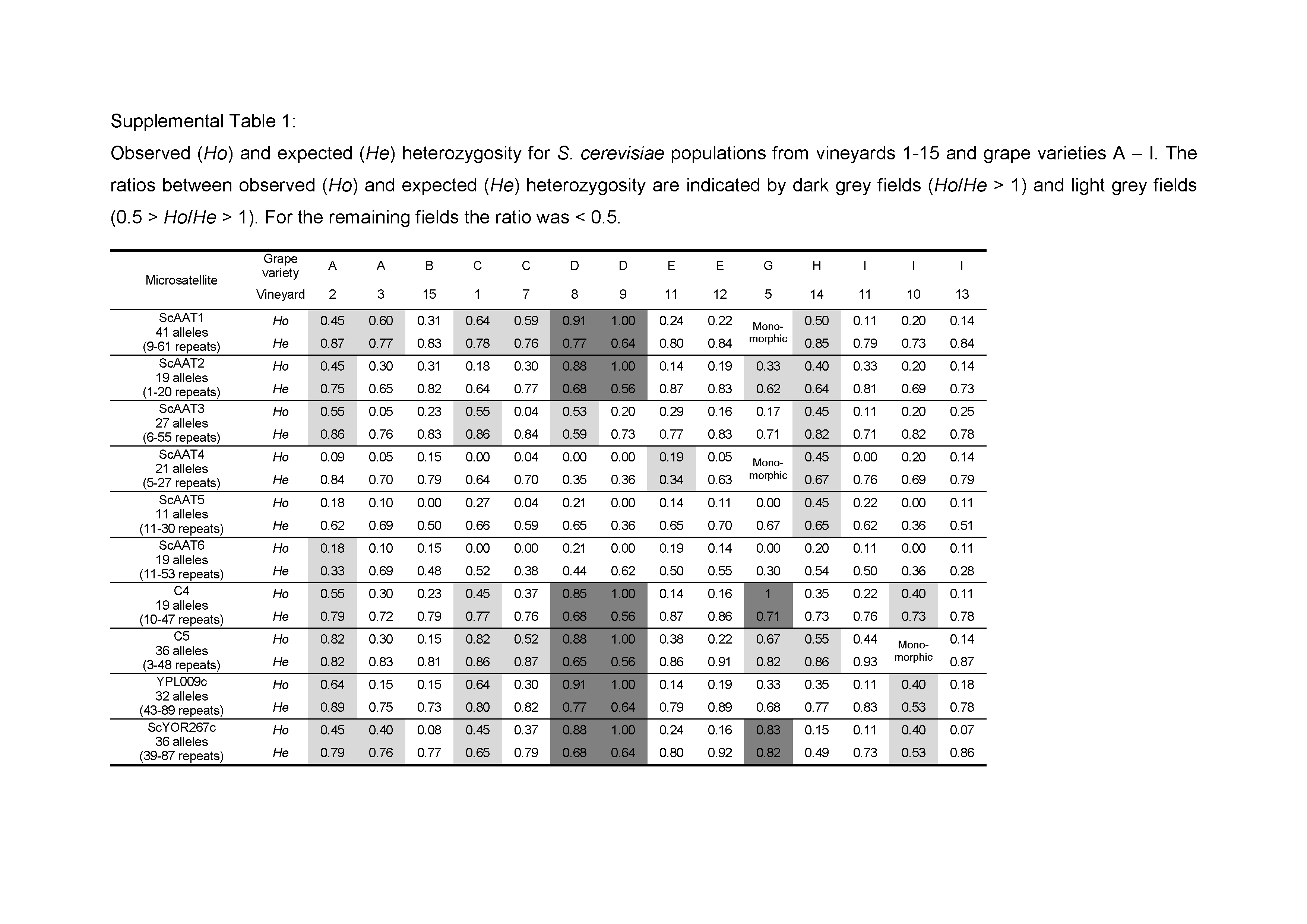

Supplement: Table S1 — Observed ( Ho ) and expected ( He ) heterozygosity for S. cerevisiae populations from vineyards 1–15 and grape varieties A–I. The ratios between observed (Ho) and expected (He) heterozygosity are indicated by underlined bold letters (Ho/He>1) and underlined letters (0.5>Ho/He>1). For the remaining fields the ratio was <0.5. (TIF) [file pone.0032507.s001.tif]
